# Antipsychotic prescribing for Alzheimer’s disease and related disorders in specialized settings from 2010 to 2014 in France: a repeated cross-sectional study

**DOI:** 10.1186/s13195-017-0256-8

**Published:** 2017-04-26

**Authors:** Karim Tifratene, Valeria Manera, Roxane Fabre, Auriane Gros, Susanne Thummler, Christian Pradier, Philippe Robert, Renaud David

**Affiliations:** 1 EA Cobtek, University of Nice Sophia-Antipolis, Nice, France; 2 Department of Public Health, L’Archet Hospital, Nice University Hospital, Nice, France; 3 Research and Resources Memory Centre, Nice University Hospital, Nice, France; 4 Present address: Centre Mémoire de Ressources et de Recherche, Institut Claude Pompidou, 10 rue Molière, 06100 Nice, France

**Keywords:** Dementia, Alzheimer’s disease, Antipsychotics, Drug prescribing, French Alzheimer Database

## Abstract

**Background:**

Safety warnings from health authorities are currently intended to limit the use of antipsychotics (APs) in dementia-related conditions to treat neuropsychiatric symptoms, such as disturbing and/or delusional behaviors. The aim of this study is to investigate prevalence, correlates and trends of AP prescribing among people with dementia between 2010 and 2014 in the French population.

**Methods:**

AP prescribing and associated factors among individuals with AD, mixed dementia and vascular dementia in the French National Alzheimer Database between 2010 and 2014 were analyzed using multivariate generalized estimating equations models (*n* = 199,549).

**Results:**

In 2014, 7.7% of people with dementia were prescribed an AP. Compared with 2010 there was a 16% increase in AP use. Multivariate analysis showed a linear increase risk of prescription with an adjusted odds ratio (95% confidence interval) of 1.23 (1.17–1.30) in 2014 compared with 2010. Factors associated with AP prescribing were male gender, more severe cognitive decline and living in long-term care facilities. Older age and higher education were protective toward AP prescribing. The type of dementia did not have any influence on AP prescribing.

**Conclusion:**

An increase in AP prescribing among individuals with dementia in French specialized settings over the last 5 years occurred despite safety warnings. This phenomenon suggests that alternative solutions for the management of behavioral and psychiatric symptoms in these populations are still urgently needed.

**Electronic supplementary material:**

The online version of this article (doi:10.1186/s13195-017-0256-8) contains supplementary material, which is available to authorized users.

## Background

Behavioral and psychological symptoms of dementia (BPSD), also known as neuropsychiatric symptoms, affect more than 90% of individuals during the course of Alzheimer’s disease (AD) [1], and are also very common in other neurodegenerative diseases. The management of BPSD is challenging and specific pharmacological and nonpharmacological approaches have been proposed [2].

Antipsychotic (AP) prescribing has frequently been recognized as a last-resort solution in order to manage disturbing symptoms such as aggression and agitation, and psychotic symptoms such as delirium and hallucinations [3]. There is a general lack of evidence about AP efficacy in older people with dementia and about risks of serious adverse events, including short and long-term increased mortality rates [4–6]. Conventional AP drugs can cause extrapyramidal syndrome and tardive dyskinesia [7]. The use of atypical AP drugs can lead to acute and subacute side effects, in particular sedation, postural hypotension and falls, especially at high doses. AP use is associated with greater severity of dementia and poorer medical status [8], more rapid decline in global cognitive level, as well as increased neuropsychiatric symptoms [9].

Despite this, AP prescribing remains very common to reduce BPSD. APs remain likely overprescribed in long-term care facilities with limited alternative resources, and many older individuals receiving APs do often not fulfill prescription criteria approved by regulation authorities’ guidelines [10]. High prevalence of AP use has been reported, in large cohorts, among older populations living in the community and in long-term care facilities, and concerning individuals diagnosed with and without dementia [10, 11].

The lack of evidence on AP efficacy in the management of BPSD and the numerous side effects translate at the regulatory approval level into no AP drug being approved for use in dementia in the USA, and only Risperidone is labeled in Europe, including France.

Excessive AP use in older populations with dementia has led several regulation authorities to publish safety warnings [11]. The impact of these warnings had been studied in different settings and most often showed a decrease, to various extents, in AP use among older individuals with and without dementia. For instance, a study in Finland reported decreased use of AP in nursing homes (between 2003 and 2011) from 42.6% to 27.8%, associated with an increase from 26.9% to 32.0% in assisted living facilities (between 2007 and 2011), with a similar trend regarding the mean number of psychotropic medications (AP, antidepressants, anxiolytics and hypnotics) [12]. In the USA, regulatory warnings regarding the use of atypical AP in the management of dementia-related conditions showed minimal impact in noninstitutionalized populations, with a compensatory shift in favor of benzodiazepines and anti-dementia medications [13]. However, another initiative in the USA allowed a 12% decrease (from 21.3% to 18.7%) in AP use in nursing homes between 2012 and 2013 [14]. In France, AP use decreased in the older population between 2003 and 2011, especially in persons with dementia (from 14.2% to 10.2%) [11]. The timing of the decrease, however, did not coincide with safety warning publications.

AP prescribing in individuals with dementia is still debated and recently the American Psychiatric Association issued guidelines focusing ‘on the judicious use of AP medications when agitation or psychosis occurs in association with dementia’ [15]. New evidence shows that an increased risk of death after AP prescribing is present for individuals with and without dementia, with a more pronounced risk in control participants without dementia than in individuals with dementia [16]. Moreover, despite most studies agreeing on the drawbacks of AP, a real benefit in the management of BPSD seems to exist for the more advanced symptomatic stages of dementia [17]. In this context, and taking into account that no major advancements in the treatment of BPSD in dementia occurred in the last decade and that practitioners have, in general, limited knowledge and/or access to alternative, nonpharmacological solutions in BPSD management [18], we hypothesized that the prevalence of use of AP prescribing in demented patients is not decreasing.

The aim of the present study was to evaluate the annual prevalence of AP prescribing in individuals with AD and related disorders in France between 2010 and 2014. We additionally explored sociodemographic and clinical factors associated with the use of AP.

## Methods

### French National Alzheimer Database (Banque Nationale Alzheimer)

The Banque Nationale Alzheimer (BNA) is part of the French strategy to fight against dementia [19–21] and records information since the end of 2009. The aim of this database is to provide information about the medical activity of French memory centers in order to adapt the healthcare provision and to generate epidemiologic knowledge on the diseases and medical practices. Information collected in the BNA consists of a limited set of data concerning demographic, diagnostic and clinical data selected by a national consensus group. The number of variables was restricted to facilitate and enhance care providers participating in this national database. Data are collected from 427 French memory units—399 memory centers (local level) and 28 resource and research memory centers (regional level)—and from 61 independent neurologists who expressed a willingness to participate.

Each time a patient consults a center, a record is generated and transferred to the database. Therefore, one patient can figure more than once in the BNA, depending on the number of medical acts he/she underwent.

Variables used for this study are: gender, age, living conditions, education, type of center, referring modalities, location of the patient, Mini Mental Score Examination (MMSE) [22], date of consultation, diagnosis and treatments. The BNA differentiates 38 diagnostic groups, based on the ICD-10 classification. Code F00.1 relates to AD, code F00.2 to mixed dementia (MD) and code F01.9 is used for vascular dementia (VD). For treatments, the BNA records the presence of a prescription at the time of the consultation for six groups of psychotropic drugs classified as follows, using ATC codes: antidepressant (N06A), anxiolytic (N05B), hypnotic (N05C), antipsychotic (N05A), cholinesterase inhibitors (ChEIs) (N06DA) and *N*-Methyl-D-aspartate receptor antagonist (NMDA antagonist) (N06DX01). No data are available on drug generics or brand names, nor on dosage. More details on this database are described elsewhere [23].

### Study design and subject selection

A repeated cross-sectional study was conducted using data from the BNA from January 1, 2010 to December 31, 2014. Only individuals who received one of the three diagnoses of interest (AD (F00.1), MD (F00.2) and VD (F01.9)) (among the 38 diagnostic groups available in the BNA) at least once during the study timeframe were included in the analysis. Individuals receiving prescriptions of anti-AD agents without an associated AD diagnosis were not considered for the analysis, as well as individuals with any other ICD-10 diagnosis such as major depressive disorder (F33), psychosis (F29) and anxiety-related disorders (F40, F41, F42, F43).

A single patient could have different entries in different years of study, corresponding to different consultations. To describe the whole population included in the study, we selected the first diagnosis attributed to the patient within the study period. We adopted the same rule to describe the population according to the year of consultation; so if an individual had two or more different diagnoses between 2010 and 2014 then the first was systematically considered. Similarly, if patients were assigned different scores for the MMSE tests during the period of interest (single year or 5-year period), the first MMSE was systematically considered for the descriptive analysis. Given the importance of cognitive status, only patients with at least one existing MMSE evaluation were considered in the analysis.

Because the event of interest for the study is drug prescribing, if an individual was prescribed an AP at any time during 1 year then this person was considered “under treatment” during this year. This status was reviewed each year.

### Statistical analysis

Descriptive analyses were conducted using percentages. Age and MMSE scores (quantitative variables) were categorized according to the distribution observed. Annual prevalence of AP prescribing was calculated as the number of patients with at least one prescription of AP during the year divided by the number of patients with one of the three diagnoses of interest seen during the same year (an individual can only appear once in the numerator and once in the denominator). The Cochrane Armitage test for trend was used to assess a linear evolution of the annual prevalence rate of AP prescriptions between 2010 and 2014.

To analyze factors associated with AP prescribing, we performed regression analyses including time-independent variables (gender, education, referring modality, diagnosis) and time-dependent variables (age, type of center, MMSE, neuroleptic, hypnotic, anxiolytic, NMDA antagonist, ChEIs, lifestyle). To account for the correlation between repeated measurements on the same individual (the same patient can have several consultations over the period, and thus several data entries), we used a logistic regression with generalized estimating equations (GEE) method.

All variables significantly associated with AP prescribing in univariate GEE models with a *p*-value threshold under 0.05 where included in multivariate GEE models. A backward stepwise elimination approach was used and only variables still significantly associated with the outcome variable with *p* < 0.05 were kept in the final model. In order to take into account an effect of time on the covariables, we tested all second-order interactions concerning the variable “year of consultation”. Significant interactions were included in the final model. Adjusted odds ratios (adj. ORs) are presented with the 95% confidence interval.

All tests were performed bilaterally. Statistical analyses were carried out with SAS Enterprise Guide software, version 5.1 (SAS Inc., Cary, NC, USA).

## Results

**Fig. 1:**
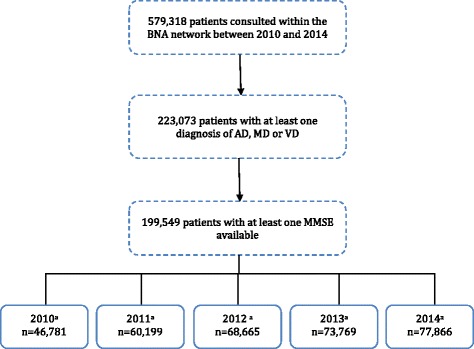
Selection of participants included in the study. ^a^The same individual could account for different years. When considering a single year, an individual can only be counted once. *AD* Alzheimer’s disease, *BNA* Banque Nationale Alzheimer, *MD* mixed dementia, *MMSE* Mini Mental State Examination, *VD* vascular dementia

Between 2010 and 2014, 223,073 individuals with a diagnosis of AD, MD or VD consulted one of the BNA centers. Among these, 199,549 individuals (mean (SD) age: 81.72 (7.50); median (IQR) age: 82.66 (78.01; 86.62)) were considered for analysis because they had at least one global cognitive evaluation using the MMSE over the period (mean of 1.7 consultations) (Fig. 1). The number of patients who consulted the BNA network increased from 40,781 patients in 2010 to 77,866 in 2014.

Demographic characteristics of the study population according to the diagnosis are presented in Table 1. The population of individuals with dementia was diagnosed mainly as AD (65.4%), followed by MD (23.7%) and VD (10.9%). Most individuals with dementia were female (66.7%), living in the community (83.9%), addressed by the GP (65.9%) and had a primary or lower education level (58.5%). Compared with AD and MD, individuals with VD appeared to more often be males, with a less advanced cognitive decline measured by MMSE and more frequently referred by a specialist.

**Table 1:** Descriptive characteristics by type of dementia

	All (*n* = 199,549)		Alzheimer disease (*n* = 130,442)		Mixed dementia (*n* = 47,320)		Vascular dementia (*n* = 21,787)	
Follow-up time (years), mean (SD)	0.87	(1.18)	0.94	(1.21)	0.83	(1.16)	0.55	(0.96)
	*n*	(%)	*n*	(%)	*n*	(%)	*n*	(%)
Year of first consultation								
2010	47,624	(23.9)	32,900	(25.2)	10,831	(22.9)	3,893	(17.9)
2011	37,236	(18.7)	24,925	(19.1)	8,565	(18.1)	3,746	(17.2)
2012	37,521	(18.8)	24,367	(18.7)	8,894	(18.8)	4,260	(19.6)
2013	37,902	(19.0)	24,175	(18.5)	9,060	(19.2)	4,667	(21.4)
2014	39,266	(19.7)	24,075	(18.5)	9,970	(21.1)	5,221	(24.0)
Age at first consultation with the diagnosis								
≤75 years	29,250	(14.7)	20,818	(16.0)	4,717	(10.0)	3,715	(17.1)
76–80 years	39,887	(20.0)	26,459	(20.3)	9,001	(19.0)	4,427	(20.3)
81–85 years	61,481	(30.8)	39,853	(30.6)	15,279	(32.3)	6,349	(29.1)
>85 years	68,931	(34.5)	43,312	(33.2)	18,323	(38.7)	7,296	(33.5)
Gender								
Female	133,102	(66.7)	91,538	(70.2)	29,493	(62.3)	12,071	(55.4)
Male	66,447	(33.3)	38,904	(29.8)	17,827	(37.7)	9,716	(44.6)
Education (years)								
0	15,955	(8.0)	10,351	(7.9)	3,750	(7.9)	1,854	(8.5)
1–5	100,745	(50.5)	64,068	(49.1)	25,527	(54.0)	11,150	(51.2)
6–12	48,163	(24.1)	31,965	(24.5)	10,809	(22.8)	5,389	(24.7)
≥13	14,358	(7.2)	9,955	(7.6)	2,932	(6.2)	1,471	(6.8)
Unknown	20,328	(10.2)	14,103	(10.8)	4,302	(9.1)	1,923	(8.8)
Type of centre								
Memory clinic	150,404	(75.4)	96,381	(73.9)	37,242	(78.7)	16,781	(77.0)
Regional specialized memory clinic	43,951	(22.0)	30,138	(23.1)	9,136	(19.3)	4,677	(21.5)
Private practice neurologist	5,194	(2.6)	3,923	(3.0)	942	(2.0)	329	(1.5)
Initially referred by								
GP	131,589	(65.9)	89,445	(68.6)	30,141	(63.7)	12,003	(55.1)
Neurologist	9,564	(4.8)	6,637	(5.1)	1,702	(3.6)	1,225	(5.6)
Other specialist	22,240	(11.2)	13,016	(10.0)	5,996	(12.7)	3,228	(14.8)
Direct	8,499	(4.3)	6,077	(4.7)	1,733	(3.7)	689	(3.2)
Others	27,657	(13.9)	15,267	(11.7)	7,748	(16.4)	4,642	(21.3)
Lifestyle at baseline								
Community-living	167,324	(83.9)	111,034	(85.1)	38,602	(81.6)	17,688	(81.2)
Long-term care facility	32,225	(16.2)	19,408	(14.9)	8,718	(18.4)	4,099	(18.8)
Location of the patient at baseline								
Within 50 km from the memory clinic	184,381	(92.4)	119,769	(91.8)	44,323	(93.7)	20,289	(93.1)
>50 km from the memory clinic	15,168	(7.6)	10,673	(8.2)	2,997	(6.3)	1,498	(6.9)
MMSE at first record								
0–10	26,451	(13.3)	19,074	(14.6)	5,585	(11.8)	1,792	(8.2)
11–20	102,300	(51.3)	66,727	(51.2)	25,916	(55.8)	9,657	(44.3)
21–30	70,798	(35.5)	44,641	(34.2)	15,819	(33.4)	10,338	(47.5)
At least one antipsychotic prescribing between 2010 and 2014								
No	181,795	(91.1)	118,685	(91.0)	43,089	(91.1)	20,021	(91.9)
Yes	17,754	(8.9)	11,757	(9.0)	4,231	(8.9)	1,766	(8.1)

*MMSE* Mini Mental State Examination

Among all diagnostic subgroups, 17,754 individuals (8.9%) were prescribed an AP at least once in the 5-year period. Annual prevalence of AP use significantly increased in a linear manner from 6.5% to 7.7% between 2010 and 2014 (*p* < 10^–5^) (Table 2). This increase was observed for community-dwelling individuals and long-term care facilities (Additional file 1).

**Table 2:** Prevalence of antipsychotic prescribing and concomitant psychotropic medications across years from 2010 to 2014

		2010		2011		2012		2013		2014	
		*n*	(%)	*n*	(%)	*n*	(%)	*n*	(%)	*n*	(%)
At least one antipsychotic prescribing											
Overall	No	43,742	(93.5)	55,898	(92.9)	63,758	(92.9)	68,369	(92.7)	71,853	(92.3)
	Yes	3,039	(6.5)	4,301	(7.1)	4,907	(7.2)	5,400	(7.3)	6,013	(7.7)
	Total	46,781		60,199		68,665		73,769		77,866	
Alzheimer’s disease	No	30,123	(93.4)	38,513	(92.9)	43,454	(93.0)	45,857	(92.8)	47,180	(92.4)
	Yes	2,146	(6.7)	2,954	(7.1)	3,276	(7.0)	3,549	(7.2)	3,871	(7.6)
	Total	32,269		41,467		46,730		49,406		51,051	
Mixed dementia	No	10,011	(93.8)	12,669	(92.6)	14,599	(92.5)	15,928	(92.5)	17,208	(92.0)
	Yes	661	(6.2)	1,006	(7.4)	1,184	(7.5)	1,299	(7.5)	1,491	(8.0)
	Total	10,672		13,675		15,783		17,227		18,699	
Vascular dementia	No	3,608	(94.0)	4,716	(93.3)	5,705	(92.7)	6,584	(92.3)	7,465	(92.0)
	Yes	232	(6.0)	341	(6.7)	447	(7.3)	552	(7.7)	651	(8.0)
	Total	3,840		5,057		6,152		7,136		8,116	
Psychotropic associations among patients receiving at least one antipsychotic prescribing											
Antipsychotic only		1,002	(33.0)	1,248	(29.0)	1,471	(30.0)	1,676	(31.0)	1,722	(28.6)
Antipsychotic + hypnotic		153	(5.0)	174	(4.1)	217	(4.4)	215	(4.0)	205	(3.4)
Antipsychotic + hypnotic + antidepressant		134	(4.4)	208	(4.8)	192	(3.9)	215	(4.0)	228	(3.8)
Antipsychotic + antidepressant		639	(21.0)	976	(22.7)	1,07	(21.8)	1,142	(21.2)	1,285	(21.4)
Antipsychotic + anxiolytic		377	(12.4)	531	(12.4)	619	(12.6)	680	(12.6)	739	(12.3)
Antipsychotic + anxiolytic + hypnotic		106	(3.5)	125	(2.9)	148	(3.0)	172	(3.2)	202	(3.4)
Antipsychotic + anxiolytic + antidepressant		463	(15.2)	767	(17.8)	895	(18.2)	982	(18.2)	1,222	(20.3)
Antipsychotic + anxiolytic + hypnotic + antidepressant		165	(5.4)	272	(6.3)	295	(6.0)	318	(5.9)	410	(6.8)
Anti-dementia agents among patients receiving at least one antipsychotic prescribing											
NMDA antagonist	No	1,756	(57.8)	2,865	(66.6)	3,556	(72.5)	3,981	(73.7)	3,933	(65.4)
	Yes	1,283	(42.2)	1,436	(33.4)	1,351	(27.5)	1,419	(26.3)	2,080	(34.6)
ChEI	No	1,433	(47.2)	1,981	(46.1)	2,588	(52.7)	3,323	(61.5)	3,443	(57.3)
	Yes	1,606	(52.9)	2,320	(53.9)	2,319	(47.3)	2,077	(38.5)	2,570	(42.7)
ChEI + NMDA antagonist	No	2,473	(81.4)	3,616	84.1	4,345	(88.6)	4,946	(91.6)	5,227	(86.9)
	Yes	566	(18.6)	685	15.9	562	(11.4)	454	(8.4)	786	(13.1)
	Total	3,039		4,301		4,907		5,400		6,013	

*ChEI* cholinesterase inhibitors, *NMDA N*-methyl-D-aspartate receptor

Considering individuals who received at least one AP prescribing, around 70% were prescribed AP with another psychotropic medication. The most frequent concomitant prescribing was AP + antidepressant with a stable proportion over the period (around 21%). A quarter of the patients were prescribed AP with two other psychotropic drugs. Among individuals receiving at least one AP medication, a third received a NMDA antagonist and half received a ChEI, with a progressive decrease from 2010 to 2013 followed by an increase as of 2014.

Table 3 presents the adj. ORs estimated by the final logistic multivariate GEE model for AP prescribing. The increase in AP prescribing between 2010 and 2014 was confirmed in multivariate analysis with an adj. OR (95% confidence interval) of 1.2 (1.17–1.30) in 2014 compared with 2010. There was a systematic significant increase of the adj. OR for the next year independently of the year of reference chosen in the model (results not shown). There was a gradual increased risk of AP prescribing with lower MMSE scores. Male gender and living in long-term care facilities were also associated with an increased risk of AP prescription. Additionally, the use of any other type of psychotropic drugs or anti-dementia agents (except for ChEIs) was an associated factor of AP prescribing. Older age and high educational levels were protective against the risk of AP prescription. Compared with individuals treated in memory clinics, individuals in regional reference memory clinics and individuals treated by private practice neurologists were less likely to receive an AP drug.

**Table 3:** Determinants of antipsychotic prescribing using multivariate logistic GEE estimation (*N* = 199,549)

	Adjusted odds ratio	95% confidence interval	*p* value
Year			
2010	1		
2011	1.10	1.05–1.15	<0.001
2012	1.12	1.06–1.17	<0.001
2013	1.16	1.10–1.21	<0.001
2014	1.23	1.17–1.30	<0.001
MMSE			
0–10	2.63	2.51–2.75	<0.001
11–20	1.56	1.51–1.62	<0.001
21–30	1		
Age at first consultation with the diagnosis			
≤75 years	1		
76–80 years	0.91	0.86–0.96	0.001
81–85 years	0.85	0.81–0.90	<0.001
>85 years	0.80	0.76–0.84	<0.001
Gender			
Female	1		
Male	1.34	1.30–1.39	<0.001
Education (years)			
0	1		
1–5	0.87	0.82–0.92	<0.001
6–12	0.84	0.78–0.89	<0.001
≥13	0.77	0.70–0.84	<0.001
Unknown	0.96	0.89–1.03	0.224
Type of centre			
Memory clinic	1		
Regional reference memory centre	0.83	0.77–0.91	<0.001
Private practice neurologist	0.60	0.42–0.86	0.006
Antidepressant			
No	1		
Yes	1.77	1.71–1.84	<0.001
Hypnotic			
No	1		
Yes	1.91	1.82–2.01	<0.001
Anxiolytic			
No	1		
Yes	2.42	2.33–2.51	<0.001
NMDA antagonist			
No	1		
Yes	1.32	1.28–1.36	<0.001
Lifestyle			
Community-living	1		
Long-term care facility	2.01	1.94–2.09	<0.001
Patient's location			
Within 50 km from memory consultation	1		
>50 km from memory consultation	0.93	0.87–1.00	0.036
Years × type of center^a^			<0.001

*GEE* generalized estimating equations, *MMSE* Mini Mental state examination, *NMDA N*-methyl-D-aspartate receptor

^a^Significant interaction entered in the model

An interaction between type of center and time was found to be significant (*p* < 0.001) and was kept in the final multivariate model. This showed that AP prescribing levels increased more in regional centers than in memory clinics over time while the use of AP did not vary significantly between 2010 and 2014 for independent neurologists. The type of dementia (AD, VD or MD) did not significantly influence AP prescribing.

## Discussion

In this large study based on the French National Alzheimer Database, the annual prevalence of AP prescribing among individuals with dementia slightly increased from 6.5% in 2010 to 7.7% in 2014 despite safety warnings issued in France (as well as in Europe and in the USA). This trend was confirmed in the multivariate analysis with a constant increased risk of prescription over time.

### Prevalence of AP use and associated factors

Our results regarding prevalence of AP use are consistent with previous studies among individuals with dementia [24–27], whereas other authors reported much higher rates of AP use [12, 28–32]. The settings of the present analysis may explain some of the observed discrepancies. First, most individuals were living in the community at a stage of the disease at which psychiatric symptoms do not yet imply long-term institutionalization, which may explain lower AP prescribing [33]. Second, the study took place in specialized settings where specialists are often able to propose nonpharmacological treatments for BPSD, and this could reduce the use of AP. This is illustrated by the fact that, in this study, an individual treated in a regional center rather than a local center has a lower chance of receiving an AP, probably because nonpharmacological approaches are more developed in reference centers. Third, several authors also included the presence of delirium or psychotic conditions as inclusion criteria likely leading to AP use. We decided not to include individuals with ‘psychotic disorders’ as a main diagnosis in our analysis whereas this ICD-10 diagnosis is also available in the BNA. Finally, the use of AP may vary across countries and different health systems, as shown in de Mauleon et al.’s study [34] investigating AP prescribing among individuals with dementia recently admitted to long-term care facilities in eight European countries, with prevalence ranging from 12% in Sweden to 54% in Spain (with an average use of 37.4%, and France at 26.5% being the second least prescribing country after Sweden).

In the present study, several factors significantly associated with AP use have been identified. One of the main factors was cognitive decline as measured here with the MMSE score and known to be associated with BPSD and with an increased risk of AP prescribing [8, 25, 35]. Younger age, male gender and institutionalization are also risk factors associated with increased AP prescribing [25]. Higher education was associated with a lower probability of AP use, with adj. OR decreasing as the educational level increased. This finding has already been reported in recent studies [36] and can have several explanatory hypotheses: a differential expression of symptoms in highly educated versus less educated individuals; a preference for nonpharmacological treatments expressed by the family of highly educated patients [37]; and, finally, the possibility of an adaptation of the physician’s attitude to the educational level of the patient and their family.

Among patients with AP prescribing, about 70% were exposed to concomitant drug prescribing. Multiple psychotropic exposures in older populations with and without dementia have been widely described [38, 39] and raise the question of the appropriateness of these concomitant uses.

The etiology of dementia did not appear to be predictive of AP use. Furthermore, we did not find any decreased risk of AP prescribing for VD, likely due to the cardiovascular side effects of AP, as described elsewhere [8].

### Evolution of AP use between 2010 and 2014

Despite the safety warnings for AP published by several regulation authorities including France in 2008 [11], we did not observe any significant decrease or stagnation in AP use in France between 2010 and 2014 among individuals diagnosed with AD, MD and VD. On the contrary, AP prescribing significantly increased to 15.6%.

The different safety warnings issued by health authorities and medical associations worldwide in the last 15 years had different effects according to the context and the period studied. Some studies reported a decrease in AP prescribing [11, 40] while other studies showed an increase or stagnation in AP use [41, 42].

Given that our results were adjusted on several confounding factors including time-dependent variables (age, global cognitive level, psychotropic drug prescribing, social context) and time-independent variables (gender, education, type of dementia), some explanations relative to the scientific and regulatory environment of prescribing could be advanced. First, no major advance in the treatment of BPSD or dementia that could change the management of the patients occurred since the warnings were issued. Moreover, in France the conditions of the reimbursement of anti-AD symptomatic agents (ChEIs and NMDA antagonists) were restricted in 2011. Anti-AD agents could contribute to the management of BPSD, and their decreased use could have promoted the use of AP. Several authors have already investigated how AP prescribing could evolve before and after the initiation of ChEIs and NMDA antagonists. For instance, Lachaine et al. [43] have shown a significant difference in AP use when comparing pre-memantine and post-memantine initiation (with a significant lower increase in AP use after memantine initiation) among individuals with AD, whereas this phenomenon was not observed with ChEIs. A similar trend was described by Martinez et al. [44], with an observed decline in AP use after memantine initiation whereas AP prescribing continued to increase after ChEI initiation. In our study, we observed a global declining use of both ChEIs and memantine during the period, with memantine only significantly associated with an increased risk of AP prescribing. Second, the way health care providers received the warning could explain their lack of long-term efficacy. For instance, a study described that professional recommendations on drugs have less impact on specialists compared with nonspecialists [45]. This hypothesis should be considered because this study took place in a specialized setting. Third, as mentioned earlier, APs have a proven efficacy to control psychotic symptoms and seem to be useful for the more symptomatic subset of people with dementia, and withdrawal of APs is not recommended in these cases [17, 46].

### Limitations of the study

Despite the size of the population and the 5 years of follow-up, several limitations should be noted.

First, the study period did not include a time before and after the date of the safety warning issued by the French Drug Agency (December 2008), so we cannot strictly assess the effect of the warning on AP prescribing. However, the trend we described is the opposite of what was expected only 2 years after the warnings. Second, the diagnoses in the BNA are entered by the physicians and reflect real life; it is thus possible that the different diagnoses are not based on the same criteria and this could introduce variability among diagnostic groups. Third, individuals included in the BNA are not fully representative of the total French population with AD and associated disorders; indeed, the BNA includes the great majority of people with AD and associated disorders who is referred to specialized centers (French memory units), but one part of the population with dementia is under GP supervision only (GPs do not have currently access to the BNA), and another part of the population is referred to specialists (geriatricians, neurologists, psychiatrists) from private practice who are not using the BNA database. Fourth, available information on AP medications in the BNA does not provide a highly complete set of information: for instance, the type of prescribed AP (i.e., first versus second generation), the drug name, the daily dose and the average treatment duration—which were all likely to change over time—were not available. Similarly, clinical reasons for and the context of treatment leading to AP prescriptions were not available in the BNA. Therefore we were not able to evaluate the appropriateness of the use of AP. Finally, we had no data on the effectiveness or tolerance of AP prescribing. We could therefore not assess whether the drug safety warnings had an impact in terms of drug-related risk minimization, which could be considered the final objective of the safety warnings rather than modifications of the prevalence of prescribing [47].

Future studies employing different databases or cohorts would be important to verify whether the observed trend of increased AP prescribing between 2010 and 2014 does extend to non-French populations with dementia, and whether similar results can be obtained with a patient population not referred to specialized memory centers. If the efficacy of safety warnings issued by health authorities in reducing AP use is controversial, some targeted educational interventions aimed at health care professionals and at a local level have shown a relative efficacy if provided regularly [14, 27].

## Conclusion

An increase in AP prescribing among individuals with dementia in French specialized settings over the last years (in a linear manner from 6.5 to 7.7% between 2010 and 2014) occurred despite safety warnings. Lower MMSE scores, male gender and living in long-term care facilities were associated with an increased risk of AP prescription. The type of dementia (AD, VD or MD) did not significantly influence AP prescribing. This increase in AP prescribing suggests that alternative solutions for the management of behavioral and psychiatric symptoms in dementia are still urgently needed.

## Additional file

Additional file 1: Prevalence of antipsychotic prescribing in the community and in long-term care facilities. (DOCX 49 kb)

## Abbreviations

AD: Alzheimer’s disease
AP: Antipsychotic
BNA: Banque Nationale Alzheimer
BPSD: Behavioral and psychological symptoms in dementia
ChEI: Cholinesterase inhibitor
GEE: Generalized estimating equations
GP: General practitioner
MD: Mixed dementia
MMSE: Mini Mental State Examination
NMDA: *N*-Methyl-D-aspartate receptor
VD: Vascular dementia
