# Supplementary material for: Antipsychotic prescribing for Alzheimer’s disease and related disorders in specialized settings from 2010 to 2014 in France: a repeated cross-sectional study
Source: Alzheimers Res Ther. 2017 Apr 26;9:34. doi: 10.1186/s13195-017-0256-8 (PMC5405526; doi:10.1186/s13195-017-0256-8)
Supplement: Additional file 1: — Prevalence of antipsychotic prescribing in the community and in long-term care facilities. (DOCX 49 kb) [file 13195_2017_256_MOESM1_ESM.docx]

|  | **2010** | | **2011** | | **2012** | | **2013** | | **2014** | |
| --- | --- | --- | --- | --- | --- | --- | --- | --- | --- | --- |
|  | **n** | **(%)** | **n** | **(%)** | **n** | **(%)** | **n** | **(%)** | **n** | **(%)** |
| **Antipsychotic prescribing** | **Community-living** | | | | | | | | | |
| No | 36918 | (94.7) | 47707 | (94.1) | 54768 | (94.2) | 58589 | (94.1) | 62237 | (93.6) |
| Yes | 2076 | (5.3) | 3002 | (5.9) | 3357 | (5.8) | 3671 | (5.9) | 4237 | (6.4) |
| **Antipsychotic prescribing** | **Long-term care facilities** | | | | | | | | | |
| No | 6824 | (87.6) | 8191 | (86.3) | 8990 | (85.3) | 9780 | (85.0) | 9616 | (84.4) |
| Yes | 963 | (12.4) | 1299 | (13.7) | 1550 | (14.7) | 1729 | (15.0) | 1776 | (15.6) |

Prevalence of antipsychotic prescribing in the community and in long-term care facilities
